# Loss of CXCR3 expression on memory B cells in individuals with long-standing type 1 diabetes

**DOI:** 10.1007/s00125-018-4651-x

**Published:** 2018-06-07

**Authors:** Wendy E. Powell, Stephanie J. Hanna, Claire N. Hocter, Emma Robinson, Joanne Davies, Gareth J. Dunseath, Stephen Luzio, Daniel Farewell, Li Wen, Colin M. Dayan, David A. Price, Kristin Ladell, F. Susan Wong

**Affiliations:** 1 Division of Infection and Immunity, Cardiff University School of Medicine, Cardiff, CF14 4XN UK; 2 Diabetes Research Unit Cymru, Swansea University, Swansea, UK; 3 Division of Population Medicine, Cardiff University School of Medicine, Cardiff, UK; 4 Section of Endocrinology, Yale University School of Medicine, New Haven, CT USA

**Keywords:** Autoimmunity, B cells, B220, BAFF, CD24, CD95, CXCL10, CXCL11, CXCR3, Type 1 diabetes

## Abstract

**Aims/hypothesis:**

Islet-specific autoantibodies can predict the development of type 1 diabetes. However, it remains unclear if B cells, per se, contribute to the causal pancreatic immunopathology. We aimed to identify phenotypic signatures of disease progression among naive and memory B cell subsets in the peripheral blood of individuals with type 1 diabetes.

**Methods:**

A total of 69 participants were recruited across two separate cohorts, one for discovery purposes and the other for validation purposes. Each cohort comprised two groups of individuals with type 1 diabetes (one with newly diagnosed type 1 diabetes and the other with long-standing type 1 diabetes) and one group of age- and sex-matched healthy donors. The phenotypic characteristics of circulating naive and memory B cells were investigated using polychromatic flow cytometry, and serum concentrations of various chemokines and cytokines were measured using immunoassays.

**Results:**

A disease-linked phenotype was detected in individuals with long-standing type 1 diabetes, characterised by reduced C-X-C motif chemokine receptor 3 (CXCR3) expression on switched (CD27^+^IgD^−^) and unswitched (CD27^intermediate^IgD^+^) memory B cells. These changes were associated with raised serum concentrations of B cell activating factor and of the CXCR3 ligands, chemokine (C-X-C motif) ligand (CXCL)10 and CXCL11. A concomitant reduction in CXCR3 expression was also identified on T cells.

**Conclusions/interpretation:**

Our data reveal a statistically robust set of abnormalities that indicate an association between type 1 diabetes and long-term dysregulation of a chemokine ligand/receptor system that controls B cell migration.

**Electronic supplementary material:**

The online version of this article (10.1007/s00125-018-4651-x) contains peer-reviewed but unedited supplementary material, which is available to authorised users.

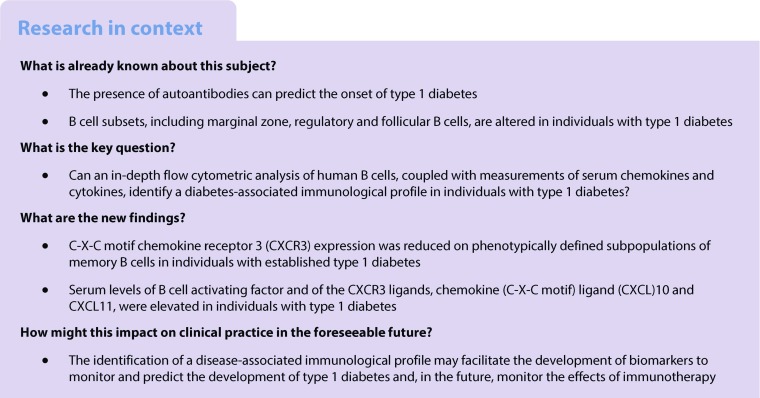



## Introduction

Type 1 diabetes is a multifactorial autoimmune disorder triggered by islet antigen-specific CD4^+^ and CD8^+^ T cell-mediated destruction of insulin-producing cells in the pancreas. Although the presence of various autoantibodies can predict the onset of type 1 diabetes, it remains unclear if the corresponding autoreactive B cells play a determinative role in the underlying immunopathology. Clinical studies have shown that B cell depletion with rituximab temporarily slows the decline of C-peptide levels in the blood of individuals with type 1 diabetes [1]. Moreover, B cells are present in the pancreatic islets at the time of diagnosis [2] and persist in situ throughout the course of disease [3].

A previous study identified transitional CD27^−^IgD^+^IgM^−^ B cell expansions in individuals with type 1 diabetes and healthy carriers of the *PTPN22* genetic variant 1858T, which predisposes to a variety of autoimmune disorders [4]. In contrast, no disease-specific alterations in the B cell compartment were detected in another study, designed to quantify the expression levels of CD19, CD24, CD27, CD38, IgD and IgM in individuals with type 1 diabetes and age- and sex-matched healthy donors [5]. Equivalent results were obtained in a comprehensive analysis of children with newly diagnosed type 1 diabetes compared with healthy control individuals [6]. However, increased frequencies of marginal zone CD19^+^CD21^+^CD23^−^ B cells and decreased frequencies of regulatory CD1d^+^CD5^+^CD19^+^ and follicular CD19^+^CD21^−^CD23^+^ B cells have been reported in Chinese individuals with type 1 diabetes [7]. Similarly, decreased frequencies of CD40^+^ and interleukin (IL)-10^+^ B cells were detected in another cohort of individuals with type 1 diabetes relative to healthy donors [8]. In addition, high-affinity insulin-binding naive B cells are lost from the anergic compartment in individuals with newly diagnosed type 1 diabetes, but return in individuals with long-standing type 1 diabetes [9]. Thus, whilst differences are present in those with type 1 diabetes compared with healthy individuals, a consistent disease-relevant phenotype in the circulating B cell pool has not been delineated.

To inform this ongoing debate, we conducted an extensive flow cytometric analysis of B cell subsets in individuals with type 1 diabetes and age- and sex-matched healthy donors.

## Methods

### Study design and setting

This study was designed to compare the phenotypes of circulating B and T cells (using flow cytometry to analyse cell-surface markers) and levels of serum chemokines and cytokines in healthy donors and people with newly diagnosed or long-standing type 1 diabetes. Venous blood samples were collected from individuals in South Wales between 2012 and 2014. Peripheral blood mononuclear cells (PBMCs) were analysed in two batches (2013 and 2014). Serum samples were cryopreserved and analysed as a single batch.

### Participants

Adults with newly diagnosed or long-standing type 1 diabetes were recruited for this study, together with age- and sex-matched healthy donors (age was matched to ±2 years). Type 1 diabetes was diagnosed according to criteria established by the American Diabetes Association [10]. Insulin treatment was commenced within 1 month of diagnosis. Time from diagnosis was categorised as less than 1 year for newly diagnosed individuals and more than 3 years for those with long-standing diabetes. Age- and sex-matched healthy donors were seronegative for islet-specific autoantibodies, with no personal or family history of type 1 diabetes or other autoimmune conditions. The discovery cohort (Study A) included *n* = 10 newly diagnosed individuals (mean age 33.4 years, range 23–44 years), *n* = 10 individuals with long-standing diabetes (mean age 40.2 years, range 29–50 years) and *n* = 15 healthy donors (mean age 38.2 years, range 24–50 years). The validation cohort (Study B) included *n* = 10 newly diagnosed individuals (mean age 24.3 years, range 19–34 years), *n* = 10 individuals with long-standing diabetes (mean age 38.5 years, range 23–48 years) and *n* = 14 healthy donors (mean age 32.5 years, range 22–48 years). Additional participants were recruited for immunoassay analysis of serum chemokines and cytokines (*n* = 34 newly diagnosed individuals; *n* = 21 individuals with long-standing diabetes; *n* = 33 healthy donors, not matched). Cohort details are summarised in electronic supplementary material (ESM) Tables 1–3.

### Ethics

This study was approved by the South East Wales Research Ethics Committee and conducted in accordance with the principles of Good Clinical Practice established by the International Council for Harmonisation and the World Health Organization. All participants provided written informed consent prior to enrolment, as mandated by the Declaration of Helsinki.

### Islet-specific autoantibodies

Serum autoantibodies specific for GAD, islet antigen-2 and zinc transporter-8 were quantified by ELISA (RSR, Cardiff, UK). Positive thresholds were set at 5 U/ml for GAD, 7.5 U/ml for islet antigen-2 and 15 U/ml for zinc transporter-8.

### HLA class II genotyping

Genomic DNA was extracted from whole blood samples collected in EDTA. *HLA-DRB* alleles (DRB1*0101-0103, DRB1*1501-1505, DRB1*1601-1606, DRB1*0301, DRB1*0401-0422, DRB1*1101-1121, DRB1*1201-1203, DRB1*1302-1305/DRB1*1303-1304, DRB1*1401/*1404*/*1405*, DRB1*0701-0702, DRB1*0805-0801, DRB1*0901, DRB1*1001, DRB3*0101-0301 and DRB4*0101) were resolved using PCRs with sequence-specific primers [11] (ESM Table 4).

### Blood samples

PBMCs were isolated from heparinised samples of whole blood via density gradient centrifugation using Lymphoprep (STEMCELL Technologies, Cambridge, UK). Aliquots of 5–20 × 10^6^ PBMCs/ml per vial were stored in liquid nitrogen after cooling overnight to −80°C at a controlled rate of −1°C/min in 90% AIM-V medium (Thermo Fisher Scientific, Hemel Hempstead, UK) supplemented with 10% dimethyl sulfoxide (Sigma-Aldrich, Gillingham, UK) (vol./vol.).

### Flow cytometry

Thawed PBMCs were blocked using TruStain (BioLegend, San Diego, CA, USA) for 5 min at room temperature, stained with LIVE/DEAD Fixable Aqua (Thermo Fisher Scientific) for 10 min at room temperature and labelled with titrated concentrations of the following monoclonal antibodies for 30 min at 4°C: α-CD19-PE-Cy7 (clone SJ25C1) and α-CD24-APC-eFluor780 (clone SN3) (both from eBioscience, San Diego, CA, USA); α-CD3-BV711 (clone OKT3), α-CD45R/B220-BV421 (clone RA3-6B2) and α-IgD-AF488 (clone IA6-2) (all from BioLegend); α-CD27-Q605 (clone CLB-27/1) (Thermo Fisher Scientific); α-CD21-PE-Cy5 (clone B-ly4), α-CD38-PE-CF594 (clone HIT2) and α-C-X-C motif chemokine receptor 3 (CXCR3)-PE (clone IC6/CXCR3) (all from BD Biosciences, Franklin Lakes, NJ, USA); and α-CD95-APC (clone DX2) (Miltenyi Biotec, Bergisch Gladbach, Germany). Cells were then washed in PBS containing 0.5% BSA (wt/vol.) and 2 mmol/l EDTA, fixed with 1% paraformaldehyde (wt/vol.) and acquired using a special-order system FACSAria II flow cytometer (BD Biosciences). Further details are available in ESM Table 5. Data were acquired in a blinded fashion and analysed using FlowJo software version 10 (Tree Star, Ashland, OR, USA).

### Spanning-tree progression analysis of density-normalised events

Live B cells were identified in serial gates as singlets, lymphocytes, LIVE/DEAD Fixable Aqua^−^, CD3^−^ and CD19^+^ events using FlowJo software version 10. Compensated flow cytometry standard files were exported and concatenated for spanning-tree progression analysis of density-normalised events (SPADE) using fixed settings (K-means algorithm, 100 clusters and arcsinh transformation with cofactor 150) in SPADE software version 3.0 (http://pengqiu.gatech.edu/software/SPADE/) [12, 13]. The following markers were included in the analysis: CD19, CD21, CD24, CD27, CD38, CD95, B220, CXCR3 and IgD. Auto-partitioning was used to divide the resulting trees into eight areas. Median expression of each marker was calculated for each donor in each node. Data from the two cohorts were tested for normality and then analysed separately because of differences in flow cytometer set-up between Study A and Study B. As the data were normally distributed, an unpaired Student’s *t* test was used to compare the transformed median fluorescence intensity (MFI) of each marker in each area for newly diagnosed individuals and those with long-standing diabetes vs healthy donors in Study A. The analysis was repeated in Study B for markers identified as significantly different in newly diagnosed individuals and those with long-standing diabetes relative to healthy donors in Study A. In a final step, B cells were gated manually, using FlowJo software version 10, to confirm differences in MFI for each marker of interest, and significance was assessed using a one-way ANOVA with Dunnett’s post hoc test.

### Serum chemokines/cytokines

Serum samples were analysed using the U-PLEX platform (Meso Scale Diagnostics, Rockville, MD, USA) to quantify chemokine (C-X-C motif) ligand (CXCL)10, CXCL11, IL-4, IL-6, IL-10 and IFN-γ or using DuoSet ELISA kits to quantify CXCL9, B cell activating factor (BAFF) and TGF-β (R&D Systems, Abingdon, UK). Data were analysed using the Kruskal–Wallis test with Dunn’s post hoc test.

### Statistics

As there were no existing data to guide power calculations, and given the large number of variables under consideration, together with the modes of analysis we planned to employ simultaneously across all groups of participants, sample sizes were determined in part by feasibility. Significant results from Study A were treated as hypothesis-generating and tested for validation purposes in Study B. An unpaired Student’s *t* test was used to assess differences in mean values between two groups.

One-way ANOVA (for normally distributed data) or the Kruskal–Wallis test (for non-normally distributed data) were used with corrections for multiple comparisons to assess differences in mean values between three or more groups. All statistical tests were performed using Prism software version 6 (GraphPad, San Diego, CA, USA).

## Results

### CXCR3 expression is reduced on memory B cells in individuals with type 1 diabetes

To evaluate the phenotypic characteristics of naive and memory B cells in individuals with type 1 diabetes, we developed a polychromatic flow cytometry panel incorporating a viability dye and monoclonal antibodies specific for CD3, CD19, CD21, CD24, CD27, CD38, CD45R/B220, CD95, CXCR3 and IgD. This panel was used to stain PBMCs from individuals with type 1 diabetes (categorised as newly diagnosed or long-standing) and healthy donors assigned randomly to separate cohorts for discovery (Study A) and validation (Study B). The data were then concatenated and analysed using SPADE.

**Fig. 1:**
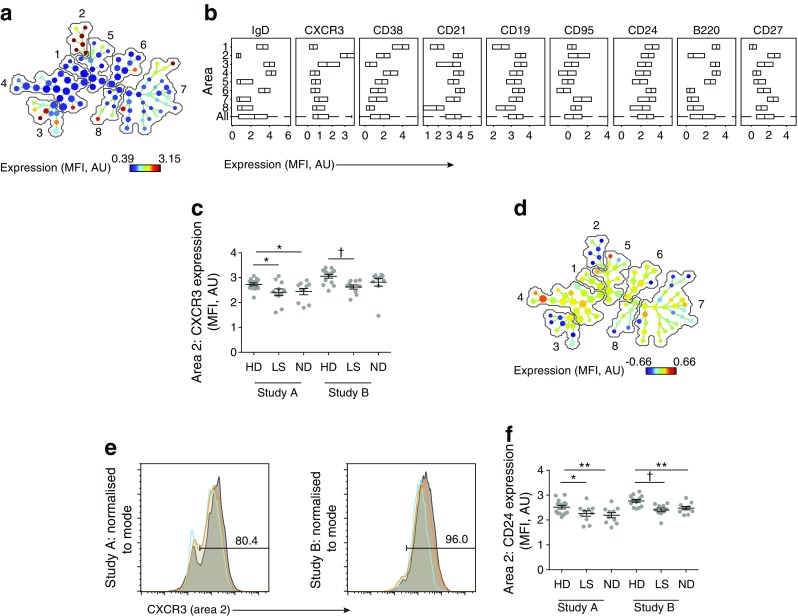
Automated phenotypic analysis of B cells. (**a**) SPADE image of pooled B cells from all participants, auto-partitioned into eight annotated areas with node size scaled to the log number of cells in each node, showing median CXCR3 expression (transformed values in SPADE using arcsinh transformation with cofactor 150) as a heatmap. Annotated areas: 1. naive (CD27^–^IgD^+^); 2. switched memory (CD27^+^IgD^–^); 3. unswitched (CD27^int^IgD^+^); 4. naive (CD27^–^IgD^+^); 5. switched (CD27^–^IgD^–^); 6. unswitched (CD27^int^IgD^+^); 7. switched memory (CD27^+^IgD^–^); 8. switched (CD27^-^IgD^–^). (**b**) Box plots of marker distribution in each area (or in all areas [All]) for the pooled samples depicted in (**a**) (central lines indicate medians, and outer lines indicate IQRs). (**c**) CXCR3 expression (transformed values) in area 2 for each individual. (**d**) SPADE image of pooled B cells from all participants, auto-partitioned into eight annotated areas (as in **a**) with node size scaled to the log number of cells in each node, showing the ratio of median CXCR3 expression (individuals with long-standing diabetes:healthy donors; transformed values) in Study B. (**e**) Histogram overlays of CXCR3 expression in area 2 for Studies A and B. Orange, newly diagnosed individuals; blue, individuals with long-standing diabetes; grey, healthy donors. (**f**) CD24 expression (transformed values) in area 2 for each individual. *p < 0.05, **p < 0.01, ^†^*p* < 0.005, Student’s *t* test. AU, arbitrary units; HD, healthy donor; ND, newly diagnosed diabetes; LS, long-standing diabetes. Means ± SEM are shown

In contrast to manual analyses of high-dimensional flow cytometry data, which may be subject to bias and user error, SPADE is a form of multivariate analysis that automatically groups closely related cells into clusters, also termed ‘nodes’, based on the expression pattern of all selected markers [14]. These clusters are reduced to a two-dimensional visual representation in which areas containing cells with similar phenotypes and marker expression are automatically partitioned. Using this approach, we identified eight phenotypically distinct B cell populations (Fig. 1a). The SPADE analysis first identified two areas of naive cells (CD27^−^IgD^+^), each of which had distinct expression of other markers (only markers that differed between the two areas are listed, with box-plots of each marker shown in Fig. 1b): area 1 cells were CD19^+^CD21^−^CD24^++^CD38^++^, and area 4 cells were CD19^++^CD21^+^CD24^+^CD38^+^. Second, there were two clusters of switched CD27^+^IgD^−^ memory cells: area 2 cells were CXCR3^++^, and area 7 cells were CXCR3^−^. Third, two further areas of switched CD27^−^IgD^−^ memory cells were identified: area 5 cells were CD19^++^CD21^+^CD38^+^CD95^−^B220^+^, and area 8 cells were CD19^+^CD21^−^CD38^−^CD95^+^B220^−^. Finally, there were two clusters of unswitched CD27^intermediate(int)^IgD^+^ B cells (area 3 cells were CD21^−/+^B220^+^CXCR3^+^, and area 6 cells were CD21^+^B220^−^CXCR3^−^). The MFI of each marker was then determined in each area for each sample (Fig. 1b), and unpaired Student’s *t* tests were used to compare area-specific differences in the expression of each marker between each diabetes group and healthy donors, with the main difference being found in CXCR3 (Fig. 1c). Non-significant results in Study A were excluded from the analysis in Study B. This strategy was adopted to minimise type 1 errors arising from multiple comparisons. Moreover, we only accepted results where a significant difference in Study A was replicated in Study B.

Statistical analysis of area 2, which comprised switched memory B cells with a CD21^+^CD24^+^CD27^+^CD38^int^CD95^+^IgD^−^ phenotype (Fig. 1a), revealed that CXCR3 expression was reduced in individuals with long-standing diabetes relative to healthy donors in Study A (*p* < 0.05) and Study B (*p* < 0.005; Fig. 1c). A similar pattern was observed for newly diagnosed individuals relative to healthy donors in Study A (*p* < 0.05; Fig. 1c), with a non-significant trend to reduced levels in Study B (*p* = 0.07). These changes were further visualised by displaying the intensity of CXCR3 expression in individuals with long-standing diabetes vs healthy donors (Fig. 1d) and by overlaying histograms of CXCR3 expression in area 2 (Fig. 1e). No significant correlation was observed between CXCR3 expression levels and the presence of HLA-DRB1*03 or HLA-DRB1*04 in healthy donors (data not shown).

**Fig. 2:**
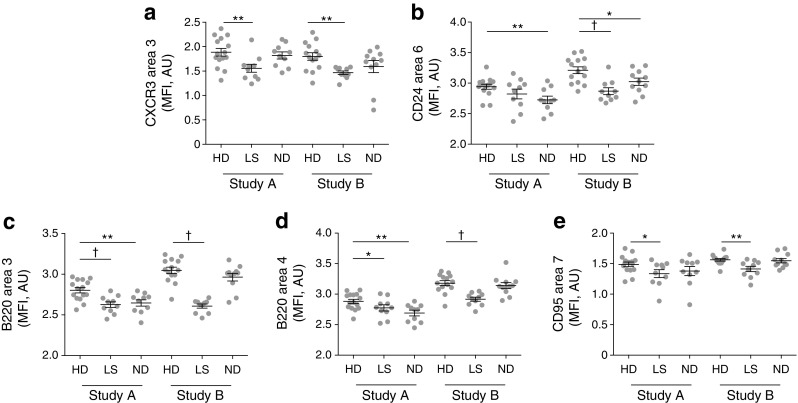
Extended phenotypic analysis of B cells. Expression levels of surface markers that differed significantly between B cell subsets in individuals with type 1 diabetes and healthy donors are shown (transformed values in SPADE using arcsinh transformation with cofactor 150) for each participant (related to Fig. 1a). (**a**) CXCR3 expression in area 3 (unswitched CD27^int^); (**b**) CD24 expression in area 6 (unswitched CD27^int^); (**c**) B220 expression in area 3 (unswitched CD27^int^); (**d**) B220 expression in area 4 (naive cells); and (**e**) CD95 expression in area 7 (switched CD27^+^ memory cells). *p < 0.05, **p < 0.01, ^†^*p* < 0.005, Student’s *t* test. AU, arbitrary units; HD, healthy donor; ND, newly diagnosed diabetes; LS, long-standing diabetes. Means ± SEM are shown

In similar analyses, we found decreased levels of CD24 expression on switched CD21^+^CD24^+^CD27^+^CD38^int^CD95^+^IgD^−^ memory B cells (area 2) in newly diagnosed individuals and those with long-standing diabetes vs healthy donors (newly diagnosed individuals: *p* < 0.01 in Study A, *p* < 0.01 in Study B; individuals with long-standing diabetes: *p* < 0.05 in Study A, *p* < 0.005 in Study B; Fig. 1f). Other significant differences were also noted across both datasets in the diabetes groups compared with healthy donors (Fig. 2). In particular, CXCR3 expression was reduced on unswitched CD27^int^IgD^+^ memory B cells (area 3) in individuals with long-standing diabetes vs healthy donors (*p* < 0.01 in Study A, *p* < 0.01 in Study B), CD45R/B220 expression was reduced on both unswitched CD27^int^IgD^+^ memory B cells (area 3: *p* < 0.005 in Study A, *p* < 0.005 in Study B) and naive CD27^−^IgD^+^ B cells (area 4: *p* < 0.05 in Study A, *p* < 0.005 in Study B), and CD95 expression was reduced on switched CD27^+^IgD^−^ memory B cells (area 7: *p* < 0.05 in Study A, *p* < 0.01 in Study B). Furthermore, in individuals with newly diagnosed diabetes, CD24 expression was reduced on unswitched CD27^int^IgD^+^ memory B cells (area 6: *p* < 0.01 in Study A, *p* < 0.05 in Study B).

**Fig. 3:**
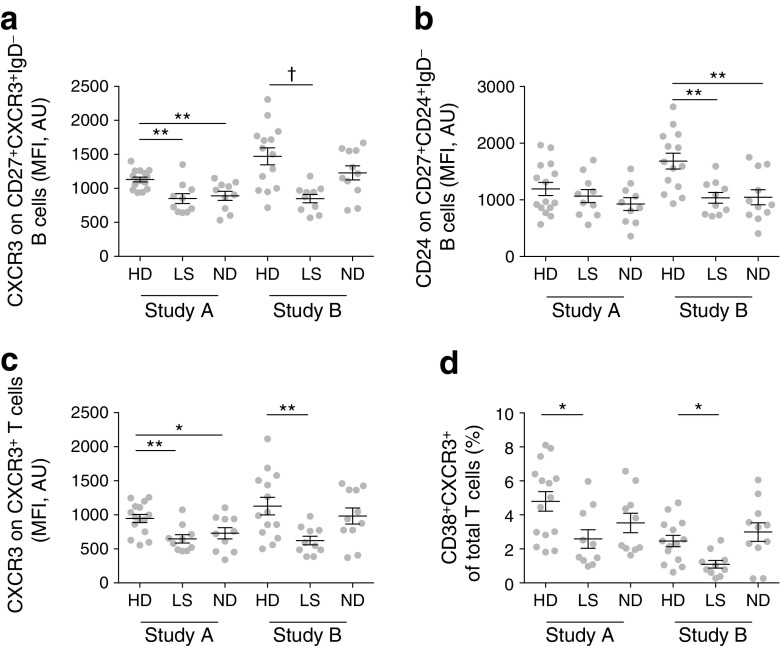
Conventional phenotypic analysis of B and T cells. Manually gated CD27^+^CXCR3^+^IgD^−^ B cells were analysed for expression of (**a**) CXCR3 and (**b**) CD24. (**c**) Manually gated CXCR3^+^ T cells were analysed for expression of CXCR3. (**d**) Manually gated CD38^+^CXCR3^+^ T cells were quantified in each individual. *p < 0.05, **p < 0.01, ^†^*p* < 0.005, one-way ANOVA with Dunnett’s post hoc test. Values are MFI calculated in FlowJo (not transformed). AU, arbitrary units; HD, healthy donor; ND, newly diagnosed diabetes; LS, long-standing diabetes. Means ± SEM are shown

To confirm these findings, we quantified expression levels of CXCR3 and CD24 on switched CD27^+^IgD^−^ memory B cells using a manual gating strategy implemented with FlowJo software (Fig. 3). A clear reduction in MFI was observed for CXCR3 expression in individuals with long-standing diabetes vs healthy donors (*p* < 0.01 in Study A, *p* < 0.005 in Study B; Fig. 3a). In contrast, CD24 expression was only reduced in Study B (*p* < 0.01; Fig. 3b).

### CXCR3 and CD38 expression are reduced on T cells in individuals with type 1 diabetes

**Fig. 4:**
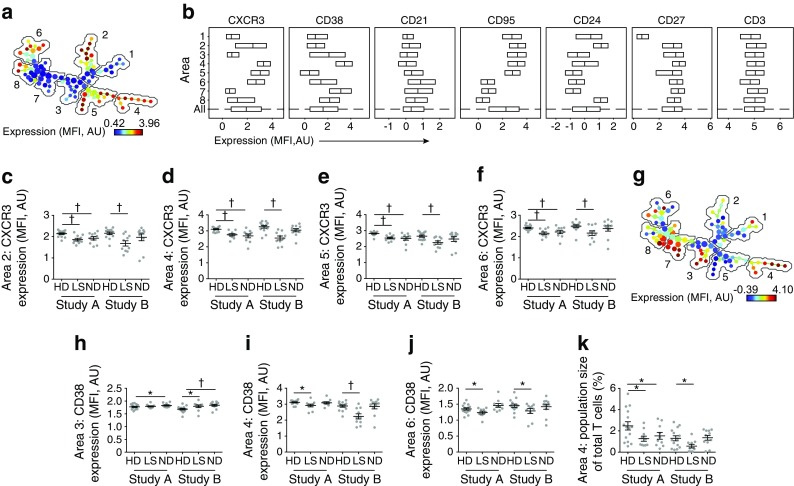
Phenotypic analysis of T cells. (**a**) SPADE image of pooled T cells from all participants, auto-partitioned into eight annotated areas with node size scaled to the log number of cells in each node (see **b** for details of each numbered area), showing median CXCR3 expression (transformed values in SPADE using arcsinh transformation with cofactor 150) as a heatmap. (**b**) Box plots of marker distribution in each area (or in all areas [All]) for the pooled samples depicted in (**a**) (the central line in the box plot indicates median, and outer lines IQR). (**c**–**f**) CXCR3 expression (transformed values in SPADE using arcsinh transformation with cofactor 150) in the indicated areas (depicted in **a**) for each participant. (**g**) SPADE image of pooled T cells from all individuals, auto-partitioned into eight annotated areas (as in **a**) with node size scaled to the log number of cells in each node, showing median CD38 expression (transformed values in SPADE) as a heatmap. (**h**–**j**) CD38 expression (transformed values in SPADE) in the indicated areas (depicted in **a**) for each participant. (**k**) Number of cells in area 4 for each participant. *p < 0.05, ^†^*p* < 0.005, Student’s *t* test. AU, arbitrary units; HD, healthy donor; ND, newly diagnosed diabetes; LS, long-standing diabetes. Means ± SEM are shown

A parallel analysis of CD3^+^ T cells revealed that CXCR3 expression was reduced in four of the eight areas identified by SPADE (Fig. 4a, b) in individuals with long-standing diabetes vs healthy donors (areas 2, 4, 5 and 6: *p* < 0.005 in Study A, *p* < 0.005 in Study B; Fig. 4c–f). High expression levels of CD38 accompanied high expression levels of CXCR3 in area 4 (Fig. 4g). In both area 4 (CD38^hi^CXCR3^hi^) and area 6 (CD38^int^CXCR3^hi^), CD38 expression was reduced in individuals with long-standing diabetes vs healthy donors (area 4: *p* < 0.05 in Study A, *p* < 0.005 in Study B; area 6: *p* < 0.05 in Study A, *p* < 0.05 in Study B; Fig. 4i, j), whereas in area 3 (CXCR3^lo^), CD38 expression was increased in newly diagnosed individuals vs healthy donors (*p* < 0.05 in Study A, *p* < 0.005 in Study B; Fig. 4h). The frequency of CD3^+^ T cells in area 4 was also lower in individuals with long-standing diabetes vs healthy donors (*p* < 0.05 in Study A, *p* < 0.05 in Study B; Fig. 4k).

These findings were confirmed using a manual gating strategy, which showed that CXCR3 expression was reduced on CD3^+^ T cells in individuals with long-standing diabetes vs healthy donors (*p* < 0.01 in Study A, *p* < 0.01 in Study B; Fig. 3c). The frequency of CD3^+^CD38^hi^CXCR3^hi^ T cells was also lower in individuals with long-standing type 1 diabetes vs healthy donors (*p* < 0.05 in Study A, *p* < 0.05 in Study B; Fig. 3d).

### CXCR3 ligand concentrations are increased in individuals with type 1 diabetes

**Fig. 5:**
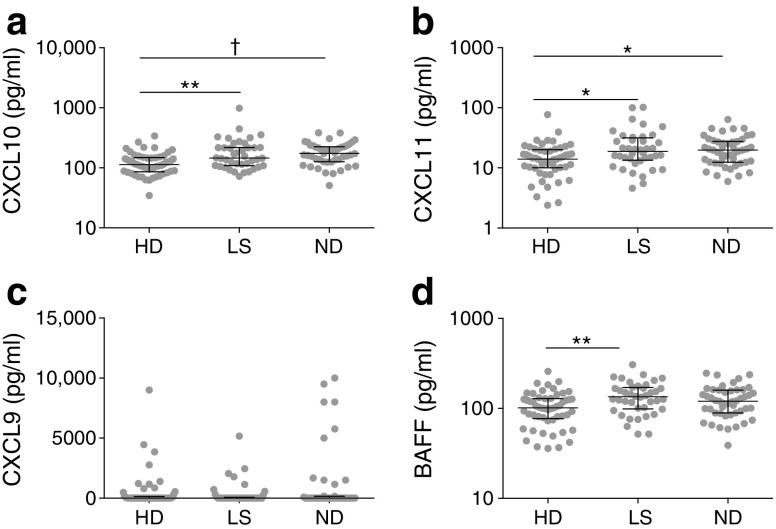
Analysis of serum chemokines and cytokines. Serum concentration of (**a**) CXCL10, (**b**) CXCL11, (**c**) CXCL9 and (**d**) BAFF for each participant. *p < 0.05, **p < 0.01, ^†^*p* < 0.005, Kruskal–Wallis test with Dunn’s post hoc test. HD, healthy donor; ND, newly diagnosed diabetes; LS, long-standing diabetes. Medians ± IQR are shown

To shed light on these phenotypic changes, we measured serum levels of the CXCR3 ligands CXCL9, CXCL10 and CXCL11, together with IL-4, IL-6, IL-10, IFN-γ, TGF-β and BAFF, which modulates the chemotactic properties of B cells to other chemokines [15, 16]. It is also notable that altered expression of the BAFF receptor has been reported in children with type 1 diabetes [15, 16]. Serum concentrations of CXCL10 and CXCL11 were significantly increased in newly diagnosed individuals and those with long-standing diabetes vs healthy donors (CXCL10: *p* < 0.005 for newly diagnosed individuals vs healthy donors, *p* < 0.01 for individuals with long-standing diabetes vs healthy donors; CXCL11: *p* < 0.05 for newly diagnosed individuals vs healthy donors, *p* < 0.05 for individuals with long-standing diabetes vs healthy donors; Fig. 5a, b). In contrast, serum concentrations of CXCL9 were generally low, with no significant differences among participant groups (Fig. 5c). Serum concentrations of BAFF were significantly increased in individuals with long-standing diabetes vs healthy donors (*p* < 0.01; Fig. 5d). There were no significant differences among groups with respect to serum concentrations of IL-4, IL-6, IL-10, IFN-γ or TGF-β (data not shown).

## Discussion

The generation of memory B cells from naive precursors is critical for the induction and maintenance of protective antibody responses to infectious agents [17]. Substantial phenotypic heterogeneity exists among memory B cells, and in various autoimmune conditions, such as rheumatoid arthritis and systemic lupus erythematosus, altered subset profiles correlate with disease activity [18]. However, few such associations have been described in individuals with type 1 diabetes.

In this study, we examined the phenotypic characteristics of naive and memory B cells in adults with type 1 diabetes and age- and sex-matched healthy donors. Our data showed that CXCR3 expression was reduced on memory B cells in individuals with long-standing diabetes. These changes were associated with raised serum concentrations of BAFF and the CXCR3 ligands CXCL10 and CXCL11. In line with previous studies, we also found that CXCR3 expression was reduced on CD3^+^ T cells in individuals with long-standing diabetes [19, 20].

Although the changes in CXCR3 expression were only significant in one of the newly diagnosed cohorts, they showed the same downward trend that we observed in the long-standing diabetes cohorts, along with the same significant increases in serum levels of CXCL10 and CXCL11. We therefore suggest that the lack of statistical significance in one of the newly diagnosed cohorts was due to a small sample size, rather than a change in CXCR3 expression limited to individuals with long-standing diabetes.

CXCR3 is constitutively expressed or readily upregulated on a substantial proportion of memory B cells [21, 22]. In contrast to individuals with rheumatoid arthritis or systemic lupus erythematosus [23], we found that expression levels of CD24 and CXCR3 on switched CD21^+^CD24^+^CD27^+^CD38^int^CD95^+^IgD^−^ memory B cells were reduced in individuals with long-standing diabetes. These cells are analogous to a highly activated population of CD21^+^CD24^+^CD27^+^CD38^+^CD95^+^CXCR3^+^IgD^−^ B cells, in which decreased expression levels of CD21 and increased expression levels of CD95 and CXCR3 correlate with activation status [18]. Moreover, they are clearly distinct from regulatory CD27^−^CD24^hi^CD38^hi^ B cells, which are defective in immune-deficient and other autoimmune conditions [5, 24, 25]. It is also notable that we did not detect increased expression levels of CD95 on switched memory B cells, as described previously in individuals with rheumatoid arthritis [26]. Instead, we found that CD95 expression was reduced on switched CD27^+^IgD^−^ memory B cells in individuals with long-standing diabetes, consistent with the findings of a recent study by Hanley and colleagues, who also reported lower frequencies of CD24^++^CD38^++^ B cells in people with type 1 diabetes [27]. This latter phenotype is indicative of regulatory B cells [28], but does not match the profiles we identified in areas 2 and 6 of the SPADE tree, where reduced expression of CD24 was apparent in newly diagnosed individuals and those with long-standing diabetes. However, it has been reported that people with asthma have decreased percentages of CD24^+^CD27^+^ B cells, which are required for the induction of IL10^+^ T cells [29]. Phenotypically, these cells would be represented in area 2 of the B cell SPADE tree, although the physiological function of the decrease in CD24 expression that we observed is currently unknown.

Serum concentrations of CXCL10 are known to be elevated in individuals with recent-onset type 1 diabetes [30–32], although there has been one report of decreased CXCL10 levels in children with type 1 diabetes [33] (reviewed in [34]). In a longitudinal study of children enrolled at diagnosis of type 1 diabetes, significantly higher CXCL10 levels were found in these participants compared with healthy control individuals [30]. Serum CXCL10 concentrations remained elevated at 1 month post diagnosis and then declined over the next 9-–30 months, persisting at levels above those detected in healthy control individuals [30]. Another study demonstrated that CXCL10 levels increase over time in at-risk individuals, peaking as they become seropositive for islet autoantibodies, irrespective of further progression to clinical type 1 diabetes [35]. In line with these earlier reports, we found increased serum concentrations of CXCL10 in adults with newly diagnosed type 1 diabetes and slightly lower levels in adults with long-standing type 1 diabetes (but elevated compared with healthy control individuals). We further demonstrated increased serum levels of CXCL11 in adults with type 1 diabetes, irrespective of time from diagnosis. Decreased expression levels of CXCR3 on memory B cells in individuals with type 1 diabetes may therefore reflect ligand-induced receptor internalisation and/or downregulation [36]. However, it has also been shown that reduced expression of CXCR3 can lead to increased ligand concentrations, as the receptor has a scavenger function [37]. An alternative possibility is that memory CXCR3^hi^ B cells traffic to the pancreas or pancreatic lymph nodes in individuals with type 1 diabetes, as shown previously for memory T cells [38, 39]. A similar phenomenon may explain why circulating switched memory B cells in individuals with type 1 diabetes also express lower levels of the adhesion molecule CD24.

CXCR3 is widely expressed in the CD3^+^ compartment, especially among T helper type 1-polarised CD4^+^ and effector CD8^+^ T cells [40]. In line with previous studies [19, 20], we found reduced expression levels of CXCR3 on circulating CD3^+^ T cells in individuals with type 1 diabetes. These findings are compatible with disease-associated trafficking of CD3^+^CXCR3^hi^ T cells to the pancreas [19, 20, 33]. In addition, we observed a simultaneous decrease in CD38 expression on a proportion of these CD3^+^ T cells. High levels of CD38 have been described on a subpopulation of peripheral CD4^+^CD25^+^CD127^dim^ regulatory T cells, which are sensitive to therapeutic intervention with the CD38-specific monoclonal antibody daratumumab in individuals with multiple myeloma [41]. It has also been reported that CD8^+^CXCR3^+^ T cells exert regulatory functions in humans [42]. Alternatively, the CD27^+^CD38^+^CD95^+^CXCR3^+^ T cell population found in area 4 may represent a stem cell-like memory (T_SCM_) subset [43]. It is notable in this regard that autoantibodies specific for CD38 have been detected in individuals with type 1 diabetes [44].

In addition to CXCL10 and CXCL11, our analysis of serum chemokines and cytokines revealed elevated concentrations of BAFF in individuals with long-standing diabetes. This cytokine plays a key role in the development of diabetes in NOD mice [45, 46], and previous studies have reported increased serum levels of BAFF in individuals with autoimmune thyroid diseases [47] and rheumatoid arthritis [48]. Decreased expression of the BAFF receptor has also been reported on B cells in children with type 1 diabetes [15]. Intriguingly, BAFF has been shown to enhance the chemotaxis of primary human B cells in response to a variety of chemokines [16]. A disease-relevant synergy may therefore exist in the setting of type 1 diabetes among raised levels of BAFF, CXCL10 and CXCL11.

Future studies will focus on investigating the ability of B cells from individuals with type 1 diabetes to migrate in response to physiological levels of CXCL10 and CXCL11, in the presence and absence of BAFF. It will be important to ascertain whether abnormal blood glucose and insulin levels in individuals with type 1 diabetes contribute to the loss of CXCR3 on memory B cells by studying the effects of ex-vivo culture and by correlating HbA_1c_ values with chemokine and chemokine receptor expression levels. It is notable in this context that glycosylation of the extracellular domains may increase signalling via CXCR3 [49]. A detailed study of postmortem pancreatic histological samples, available from the nPOD collection, may allow the identification of CXCR3^++^ B cells that trafficked to the pancreas during life, and were thus lost from the periphery. Similarly, the source of raised CXCR3 ligands in the serum remains unknown. Although there is some evidence that the inflamed pancreas can produce CXCR3 ligands in individuals with type 1 diabetes [39], it is also possible that these chemokines are produced in response to elevated glucose levels in the periphery, an effect that could be determined by longitudinal measurements of chemokine levels in conjunction with measurements of HbA_1c_.

In conclusion, we have identified a mechanistically cohesive immune profile associated with type 1 diabetes. The key abnormalities suggest long-term disruption of a chemokine ligand/receptor system that controls B cell migration. On this basis, we propose that related parameters may find utility as cellular and/or soluble biomarkers to monitor and/or predict the development of islet-specific autoantibody responses.

## Electronic supplementary material

ESM Tables (PDF 304 kb)

## Data Availability

The datasets generated and/or analysed during the current study are available from the corresponding author on reasonable request.

## Abbreviations

BAFF: B cell activating factor
CXCL: Chemokine (C-X-C motif) ligand
CXCR3: C-X-C motif chemokine receptor 3
IQR: Interquartile range
MFI: Median fluorescence intensity
PBMC: Peripheral blood mononuclear cell
SPADE: Spanning-tree progression analysis of density-normalised events
