# Supplementary material for: Loss of CXCR3 expression on memory B cells in individuals with long-standing type 1 diabetes
Source: Diabetologia. 2018 Jun 7;61(8):1794–803. doi: 10.1007/s00125-018-4651-x (PMC6061155; doi:10.1007/s00125-018-4651-x)
Supplement: Supplementary file 1 — (PDF 304 kb) [file 125_2018_4651_MOESM1_ESM.pdf]

ESM table 1

Demographics of HLA typing and serum antibody typing in patients with type 1 diabetes

				Serum antibodies U/ml			HLA Class II genotyping			
**I.D.**	**Study**	**Gender**	**Age**	**GAD**	**ZnT8**	**IA2**	***DRB1* allele**		***DRB3***	***DRB4***
LS01	A	m	29	99.8	792	520.2	*0301	-	*0101-0301	-
LS02	A	m	34	2	12	1.3	*0301	*0401-0422	*0101-0301	*0101
LS03	A	m	36	42.2	0.9	1.8	*0401-0422	-	-	*0101
LS04	A	m	44	>2000	7	3	*0401-0422	-	-	*0101
LS05	A	m	48	644.2	14.8	2.2	*0401-0422	-	-	*0101
LS06	A	f	32	245.1	16.2	1.5	*0101-0103	*0301	*0101-0301	-
LS07	A	f	36	<1	7	21	*0301	*0401-0422	-	*0101
LS08	A	f	44	1783.2	247.7	167.2	*0401-0422	-	-	*0101
LS09	A	f	49	10.2	2.8	3.3	*0301	*0401-0422	-	*0101
LS10	A	f	50	141.3	4.9	1.3	*0301	*0401-0422	-	*0101
LS11	B	m	45	15.8	7.3	4.1	*0401-0422	*0701-0702	-	*0101
LS12	B	m	46	18	136	48	*0301	*0401-0422	*0101-0301	*0101
LS13	B	m	47	93	4	2	*1601-1606	*0401-0422	-	*0101
LS14	B	m	48	124	783	152	*0301	*0401-0422	*0101-0301	*0101
LS15	B	f	23	1512.8	0.6	10.2	*0401-0422	*0805-0801	*0101-0301	*0101
LS16	B	f	23	<1	4	1	*0301	*0401-0422	-	*0101
LS17	B	f	33	128.8	2.2	1.5	*0401-0422	*1302-1305/1303-1304	-	*0101
LS18	B	f	37	3.9	1.7	0.5	*0401-0422	*1101-1121	*0101-0301	*0101
LS19	B	f	39	<1	13	1	*0301	*0401-0422	-	*0101
LS20	B	f	44	>2000	1.1	0.8	*0301	-	-	-

				Serum antibodies U/ml			HLA Class II genotyping			
**I.D.**	**Study**	**Gender**	**Age**	**GAD**	**ZnT8**	**IA2**	***DRB1* allele**		***DRB3***	***DRB4***
ND01	A	m	24	280.1	4.8	1.2	*0101-0103	*0701-0702	-	*0101
ND02	A	m	27	1249	1312	5	*0301	-	-	-
ND03	A	m	32	284	1605	528	*0301	*0701-0702	-	*0101
ND04	A	m	33	19	1058	113	*0101-0103	-	-	-
ND05	A	m	34	244.6	0.9	1.5	*0301	*0701-0702	-	*0101
ND06	A	m	38	>2000	1054	2	*0101-0103	*0301	-	-
ND07	A	f	23	242.6	226	6.8	*0401-0422	*1302-1305/1303-1304	-	*0101
ND08	A	f	36	1726	5	592	*0301	*0701-0702	-	*0101
ND09	A	f	43	1705	13	20	*0301	*0401-0422	-	*0101
ND10	A	f	44	319	NSA	10	*0101-0103	*0901	-	*0101
ND11	B	m	19	84	172	583	*0301	*0401-0422	-	*0101
ND12	B	m	19	751	1939	12	*0301	-	-	-
ND13	B	m	20	<1	23	473	*0301	*0401-0422	-	*0101
ND14	B	m	21	114	5	2	*0401-0422	*1302-1305/1303-1304	-	*0101
ND15	B	m	23	<1	160	2	*0301	*0401-0422	-	*0101
ND16	B	m	34	1764	1220	62	*1501-1505	*0301	-	-
ND17	B	m	34	<1	6	3	*0301	-	*0101-0301	-
ND18	B	f	22	33	1381	482	*0101-0103	*0701-0702	-	*0101
ND19	B	f	23	50	83	6	*0101-0103	-	-	-
ND20	B	f	28	1734.2	909	163.2	*0401-0422	*1302-1305/1303-1304	*0101-0301	*0101

LS' and 'ND' denote patients with 'long-standing' or 'newly diagnosed' type 1 diabetes respectively.
NSA, no serum available.
Cut-off for autoantibody positivity GAD, <5U/ml; IA2, <7.5 U/ml; ZnT8, <15 U/ml.

ESM table 2

Demographics of HLA typing in healthy donors

				HLA Class II genotyping			
**I.D.**	**Study**	**Gender**	**Age**	***DRB1* allele**		***DRB3***	***DRB4***
HC01	A	m	24	*0701-0702	-	-	*0101
HC02	A	m	28	*0401-0422	*0701-0702	-	*0101
HC03	A	m	33	*1501-1505	*0401-0422	-	*0101
HC04	A	m	33	*0101-0103	-	-	-
HC05	A	m	33	*0101-0103	*0701-0702	-	*0101
HC06	A	m	37	*1401,1404,1405	*0701-0702	*0101-0301	*0101
HC07	A	m	38	*1201-1203	*1302-1305/1303-1304	*0101-0301	-
HC08	A	m	43	*0301	*0805-0801	-	-
HC09	A	m	48	*0701-0702	*0901	-	*0101
HC10	A	f	34	*0301	*1302-1305/1303-1304	-	-
HC11	A	f	36	*0701-0702	-	-	*0101
HC12	A	f	43	*0101-0103	-	-	-
HC13	A	f	43	*1501-1505	-	-	-
HC14	A	f	50	*0401-0422	*1101-1121	*0101-0301	*0101
HC15	A	f	50	*0401-0422	*0701-0702	-	*0101
HC16	B	m	22	*0401-0422	-	-	*0101
HC17	B	m	24	*1101-1121	*1601-1606	*0101-0301	-
HC18	B	m	32	*0701-0702	*1001	-	*0101
HC19	B	m	43	*0301	*0805-0801	-	-
HC20	B	m	48	*0701-0702	*0901	-	*0101
HC21	B	f	25	*0101-0103	*0401-0422	-	*0101
HC22	B	f	26	*0401-0422	*0701-0702	-	*0101
HC23	B	f	26	*1401,1404,1405	-	*0101-0301	-
HC24	B	f	27	*0301	*1302-1305/1303-1304	*0101-0301	-
HC25	B	f	29	*1101-1121	*0701-0702	*0101-0301	*0101
HC26	B	f	37	*0101-0103	*0401-0422	-	*0101
HC27	B	f	34	*0301	*1302-1305/1303-1304	-	-
HC28	B	f	40	*1501-1505	*1101-1121	*0101-0301	-
HC29	B	f	43	*0101-0103		-	-

All healthy donors (HD) tested negative for antibodies.
Cut-off for autoantibody positivity GAD, <5 U/ml; IA2, <7.5 U/ml; ZnT8, <15 U/ml

ESM table 3

Demographics of additional individuals recruited for immunoassay analysis of serum chemokines/cytokines

Group	Average age	Age range	Males	Females
HD	33	18-49	19	14
LS	35	18-54	9	12
ND	27	18-50	20	14

ESM table 4 HLA Class II Genotyping Primers

Gene	Primer Name	Sequence (5'-3')	Product size (bp)	HLA-Alleles
DR1	5'01	TTG TGG CAG CTT AAG TTT GAA T	255	*DRB1*0101-0103*
	3'047	CTG CAC TGT GAA GCT CTC AC		
	3'048	CTG CAC TGT GAA GCT CTC CA		
DR15	5'02	TCC TGT GGC AGC CTA AGA G	197	*DRB1*1501-1505*
	3'01	CCG CGC CTG CTC CAG GAT		
DR16	5'02	TCC TGT GGC AGC CTA AGA G	213	*DRB1*1601-1606*
	3'02	AGG TGT CCA CCG CGG CG		
DR3	DR3 F	GTT TCT TGG AGT ACT CTA CGT C	approx 200	*DRB1*0301*
	DR3 R	TGC AGT AGT TGT CCA CCC G		
DR4	5'04	GTT TCT TGG AGC AGG TTA AAC A	250	*DRB1*0401-0422*
	3'047	CTG CAC TGT GAA GCT CTC AC		
	3'048	CTG CAC TGT GAA GCT CTC CA		
DR11	5'05	GTT TCT TGG AGT ACT CTA CGT C	176	*DRB1*1101-1121*
	3'06	CTG GCT GTT CCA GTA CTC CT		
DR12	5'08	AGT ACT CTA CGG GTG AGT GTT	248	*DRB1*1201-1203*
	3'08	CAC TGT GAA GCT CTC CAC AG		
DR13	5'03	TAC TTC CAT AAC CAG GAG GAG A		
	5'05	GTT TCT TGG AGT ACT CTA CGT C	130	*DRB1*1302-1305*
	3'10	CCC GCT CGT CTT CCA GGA T		
	3'045	TGT TCC AGT ACT CGG CGC T	171	*DRB1*1303-1304*
	3'17	CCC GCC TGT CTT CCA GGA A		
DR14	5'05	GTT TCT TGG AGT ACT CTA CGT C	224/215	*DRB1*1401,1404,1405*
	5'08	AGT ACT CTA CGG GTG AGT GTT		
	3'11	TCT GCA ATA GGT GTC CAC CT		
DR7	5'07	CCT GTG GCA GGG TAA GTA TA	232	*DRB1*0701-0702*
	3'079	CCC GTA GTT GTG TCT GCA CAC		
DR8	5'08	AGT ACT CTA CGG GTG AGT GTT	214	*DRB1*0805-0801*
	3'045	TGT TCC AGT ACT CGG CGC T		
	3'18	GCT GTT CCA GTA CTC GGC AT		
DR9	5'09	GTT TCT TGA AGC AGG ATA AGT TT	235	*DRB1*0901*
	3'079	CCC GTA GTT GTG TCT GCA CAC		
DR10	5'10	CGG TTG CTG GAA AGA CGC G	206	*DRB1*1001*
	3'047	CTG CAC TGT GAA GCT CTC AC		
DR52	5'52.1	TTT CTT GGA GCT GCG TAA GTC	171/173	*DRB3*0101-0301*
	5'52.2	GTT TCT TGG AGC TGC TTA AGT C		
	3'13	CTG TTC CAG GAC TCG GCC A		
	3'14	GCT GTT CCA GTA CTC GGC AT		
DR53	5'53	GAG CGA GTG TGG AAC CTG A	213	*DRB4*0101*
	3'048	CTG CAC TGT GAA GCT CTC CA		
Control	5'C	TGC CAA GTG GAG CAC CCA A	796	THIRD INTRON - *DRB1*
	3'C	GCA TCT TGC TCT GTG CAG AT		

ESM table 5: Flow cytometry antibody staining panel

Antibody	Fluorochrome	µl/test	Company	clone
CD19	PE-Cy7	1.5	eBioscience	SJ25C1
CD3	BV711	1	Biolegend	OKT3
IgD	AF488	2	Biolegend	IA6-2
CD27	Q605	0.5	Life Technologies	CLB-27/1
CD21	PE-Cy5	5	BD Pharmingen	B-ly4
CD24	APC eFluor780	3	eBioscience	SN3
CD38	PE CF594	0.8	BD Pharmingen	HIT2
CXCR3/CD183	PE	15	BD Pharmingen	IC6/CXCR3
CD95	APC	3	Miltenyi	DX2
CD45/B220	BV421	2.5	Biolegend	RA3-6B2

Note: ESM table 5 details, for illustration purposes only, the monoclonal antibody panel used in this study. Each antibody was titrated before use to ensure an optimal signal-to-noise ratio with our flow cytometer set-up.
